# Neural Connectivity in Syntactic Movement Processing

**DOI:** 10.3389/fnhum.2019.00027

**Published:** 2019-02-13

**Authors:** Eduardo Europa, Darren R. Gitelman, Swathi Kiran, Cynthia K. Thompson

**Affiliations:** ^1^Roxelyn and Richard Pepper Department of Communication Sciences and Disorders, Northwestern University, Evanston, IL, United States; ^2^Advocate Lutheran General Hospital, Park Ridge, IL, United States; ^3^Department of Medicine, Rosalind Franklin University of Medicine and Science, North Chicago, IL, United States; ^4^The Ken and Ruth Davee Department of Neurology Department of Neurology, Feinberg School of Medicine, Northwestern University, Chicago, IL, United States; ^5^College of Health & Rehabilitation Sciences, Boston University, Boston, MA, United States; ^6^Mesulam Cognitive Neurology and Alzheimer's Disease Center, Feinberg School of Medicine, Northwestern University, Chicago, IL, United States

**Keywords:** syntactic movement, non-canonical sentences, sentence comprehension, functional magnetic resonance imaging, dynamic causal modeling

## Abstract

Linguistic theory suggests non-canonical sentences subvert the dominant *agent-verb-theme* order in English via displacement of sentence constituents to argument (NP-movement) or non-argument positions (wh-movement). Both processes have been associated with the left inferior frontal gyrus and posterior superior temporal gyrus, but differences in neural activity and connectivity between movement types have not been investigated. In the current study, functional magnetic resonance imaging data were acquired from 21 adult participants during an auditory sentence-picture verification task using passive and active sentences contrasted to isolate NP-movement, and object- and subject-cleft sentences contrasted to isolate wh-movement. Then, functional magnetic resonance imaging data from regions common to both movement types were entered into a dynamic causal modeling analysis to examine effective connectivity for wh-movement and NP-movement. Results showed greater left inferior frontal gyrus activation for *Wh* > *NP-movement*, but no activation for *NP* > *Wh-movement*. Both types of movement elicited activity in the opercular part of the left inferior frontal gyrus, left posterior superior temporal gyrus, and left medial superior frontal gyrus. The dynamic causal modeling analyses indicated that neither movement type significantly modulated the connection from the left inferior frontal gyrus to the left posterior superior temporal gyrus, nor vice-versa, suggesting no connectivity differences between wh- and NP-movement. These findings support the idea that increased complexity of wh-structures, compared to sentences with NP-movement, requires greater engagement of cognitive resources via increased neural activity in the left inferior frontal gyrus, but both movement types engage similar neural networks.

## Introduction

Auditory sentence comprehension requires the rapid integration of phonological, semantic, and syntactic information and is primarily supported by a network of regions in the left perisylvian cortex. Neurocognitive models suggest that the left inferior frontal and posterior temporal areas are integral for sentence processing, but their functions and neural dynamics are not clearly understood. Sentence comprehension is affected by syntactic structure, in that canonical forms that follow the basic word order of a particular language (e.g., subject-verb-object (SVO) in English), as in (1) below, are easier to understand than non-canonical forms, as in (2) and (3), that deviate from canonical order. Further, there are several types of non-canonical sentences that engage unique linguistic processes that may engage differential neural networks.

1. The woman weighed the boy (Active sentence; canonical).
2. *The boy*_i_ was weighed *(t*_i_*)* by the woman (Passive sentence; non-canonical; NP-movement).
3. It was *the boy*_j_ *who*_i, j_ the woman weighed *(t*_i_*)*. (Object-cleft sentence; non-canonical; Wh-movement).

Based on linguistic theory (i.e., Government and Binding Theory Chomsky, 1986, 1995) (2) passive, and (3) object-cleft structures involve differing movement operations: NP- and wh-movement, respectively. NP-movement refers to noun phrase movement, whereas, wh-movement refers to movement of a wh-operator (e.g., *who*). In both structures, the moved constituent originates in the object position, assigned a theme by the verb, and once moved, a trace (*t*) is left behind marking its original position. In (2) the displaced theme occupies an argument (i.e., the subject) position in the sentence. However, in (3) the theme moves to a non-argument position. In both movement types, the displaced element has a dependency relationship with the trace (as noted by the subscript *i*). In addition, because object-clefts involve an embedded clause, a co-referential relation between the moved element and the head noun of the relative clause is required (denoted by the subscript *j*). This additional dependency renders the wh-movement structure in (3) more complex than the NP-movement structure in (2).

Psycholinguistic studies have examined whether these representational descriptions are associated with measurable cognitive processing costs. Cross-modal priming tasks and visual world eyetracking studies have shown increased processing time at the trace site while listening to NP- and wh-movement structures (Nagel et al., 1994; Lee, 2004; Dickey et al., 2007; Dickey and Thompson, 2009). Findings from individuals with agrammatic aphasia suggest that the double dependency in wh-movement engenders greater processing resources (Mauner et al., 1993; Dickey and Thompson, 2004; Salis and Edwards, 2005).

Functional imaging studies also have investigated the neural mechanisms of wh- and NP-movement sentences, though none have made any direct comparisons. Studies of wh-movement commonly reported activation in and around the left inferior frontal gyrus (IFG) and temporoparietal junction (TPJ) (e.g., Caplan et al., 1999, 2008; Ben-Shachar et al., 2003, 2004; Thompson et al., 2010b; Bornkessel-Schlesewsky and Schlesewsky, 2013). Not only are these regions involved with processing complex verb argument structure (Ben-Shachar et al., 2003; Thompson et al., 2010a), but they also are engaged for integrating semantic and syntactic information. Cross-linguistic studies have reported similar regions. A Hebrew study by Shetreet and Friedmann (2014) found that, when directly compared to verb movement, wh-movement elicited activity in the left IFG (BA 44/45), left posterior temporal cortex (BA 22), and medial superior frontal gyrus. Similarly, a study by Makuuchi et al. (2012) found that activity in the left pars opercularis of the IFG (BA 44) was positively correlated with distance in German sentences with scrambling, but not in those with wh-movement. Taken together, these results are in line with theories that the left posterior IFG may be involved with processes that occur after initial phrase structure building and semantic interpretation via syntactic working memory processes (Caplan et al., 1999, 2008) which precede thematic role re-analysis in the left TPJ.

Fewer studies have examined the neural correlates of NP-movement. A study comparing passive sentences to active sentences linked neural activity in the left pars opercularis and triangularis of the IFG to NP-movement and/or non-canonical verb-argument structure mapping, and activity in the left posterior middle temporal gyrus and superior parietal lobule to thematic mapping and re-analysis processes (Mack et al., 2013). These results are in line with several Japanese and Chinese NP-movement studies that also compared passive and active structures. However, it should be noted that unlike Japanese and English passives which are marked by an additional morpheme in the verb, the Chinese language has no morphological inflections (Yokoyama et al., 2007; Feng et al., 2015). Temporoparietal activity was reported in the left posterior superior temporal gyrus (STGp) (Kinno et al., 2008; Hirotani et al., 2011) for Japanese and Chinese passives, and the left superior parietal lobule (Yokoyama et al., 2006) and left inferior parietal lobule (IPL) (Yokoyama et al., 2007) for Japanese passives only–areas which have all been previously implicated in thematic re-analysis and verb-argument integration (Thompson and Meltzer-Asscher, 2014). This interpretation is further supported by an Italian study that demonstrated improved accuracy on the comprehension of passive sentences after transcranial magnetic stimulation in the left posterior parietal cortex (Finocchiaro et al., 2015). NP-movement was also associated with the left pars triangularis of the IFG for both Japanese (Yokoyama et al., 2006; Hirotani et al., 2011) and Chinese passives (Ye and Zhou, 2009), and the left pars orbitalis of the IFG in Chinese passives (Feng et al., 2015). In contrast, a few studies comparing passives to actives reported activation only in non-traditional language areas such as the left frontal operculum, caudal to the IFG (Yokoyama et al., 2007), and the postcentral gyrus (Matchin and Hickok, 2016). In summary, converging evidence across methods and languages provide support for the neural instantiation of syntactic movement which may be supported by a left hemisphere network including the TPJ and the IFG.

Two neurocognitive models of auditory sentence comprehension offer different predictions for how syntactic movement might be processed. The model by Friederici (2012) proposes that initial and higher-order syntactic processes elicit neural activity along temporo-frontal ventral and fronto-temporal dorsal pathways, respectively. The ventral tract consists of the extreme capsule fiber system and the uncinate fasciculus, and is associated with retrieval of lexical-semantic information in the middle temporal gyrus, followed by first-pass syntactic and semantic parsing in the anterior temporal lobe and anterior IFG. The dorsal tract includes the superior longitudinal/arcuate fasciculus which is involved in processing syntactic complexity. In this model, non-canonical sentences with syntactic movement first undergo phrase structure building in the left IFG, then thematic role re-analysis in the left TPJ (also see Thompson and Meltzer-Asscher (2014) for a similar model for processing verb argument structure).

On the contrary, the model proposed by Bornkessel-Schlesewsky and Schlesewsky (2013) suggests that all sentences, regardless of complexity, begin with lexical processing in the left posterior superior temporal cortex followed by temporo-frontal ventral and dorsal projections to the left frontal cortex for integration of linguistic information. In their model, the ventral tract is engaged by combinatorial semantic processes, while the dorsal tract subserves the identification and parsing of syntactic relations. These two tracts converge on the left IFG where semantics and syntax are integrated. However, this model makes two controversial claims: First, the authors argue that there are no specialized mechanisms for syntactic complexity, as they claim there is no cross-linguistic operational definition that differentiates simple from complex syntax across studies (Schlesewsky and Bornkessel-Schlesewsky, 2013). Second, they argue that the IFG is not directly involved in linguistic processing, adding to the extensive debate over the role of the left IFG in language-specific vs. domain-general functions (Hagoort, 2005; Costafreda et al., 2006; Grodzinsky and Santi, 2008; Rogalsky and Hickok, 2011). These authors associate the IFG with cognitive control and/or conflict resolution. According to their model, non-canonical sentences with syntactic movement are processed in a similar manner to canonical sentences: both engage left dorsal and ventral temporo-frontal perisylvian pathways from the posterior superior temporal cortex to the IFG.

The purpose of this study was two-fold: (1) to identify the network of regions associated with complex sentence comprehension in cognitively healthy adults, and (2) to explore how syntactic complexity modulates connectivity within this network. We operationally defined complex sentences as those with arguments in non-canonical order as a result of wh- or NP-movement. Our study used dynamic causal modeling on fMRI data acquired during an auditory sentence-picture verification task to assess the neural mechanisms of processing non-canonical structures with wh- vs. NP-movement. The first hypothesis was that operations involved with processing non-canonical sentences with wh- or NP-movement engage left perisylvian neural networks. Given previous findings of shared syntactic movement processes, we expected to see activity in the left IFG and TPJ. We also predicted that wh- and NP-movement would engage differential activation, reflecting distinct processes, in more focal regions within the left fronto-temporal network. We tested the two aforementioned models of auditory sentence processing which had competing hypotheses for how syntactic movement is processed: via a left fronto-temporal dorsal pathway (Friederici, 2012) or via left temporo-frontal dorsal and ventral pathways (Bornkessel-Schlesewsky and Schlesewsky, 2013).

## Materials and Methods

### Participants

Twenty-one participants (9 females) were recruited from Chicago and surrounding areas to participate in the study and used in the MRI analysis. They were 24–67 years of age (*M* = 36.3; *SD* = 13.1) and had an average of 18.4 years of education (*SD* = 2.5). All participants were right-handed, native speakers of English with normal or corrected-to-normal hearing and vision, and did not have a history of neurological, speech, language, or learning problems. Data from 15 of the 21 participants were used in the connectivity analysis (7 females; age in years: *M* = 33.6, *SD* = 10.8; education in years: *M* = 17.9, *SD* = 2.5) after applying additional exclusionary criteria (see Node Specification section in Effective Connectivity Analysis for details).

All participants passed a MRI safety screening and were compensated for their participation. This study was carried out in accordance with the recommendations of the Human Research Protection Program Plan, Northwestern University with written informed consent from all subjects. All subjects gave written informed consent in accordance with the Declaration of Helsinki. The protocol was approved by the Institutional Review Board at Northwestern University.

### Procedure

**Figure 1:**
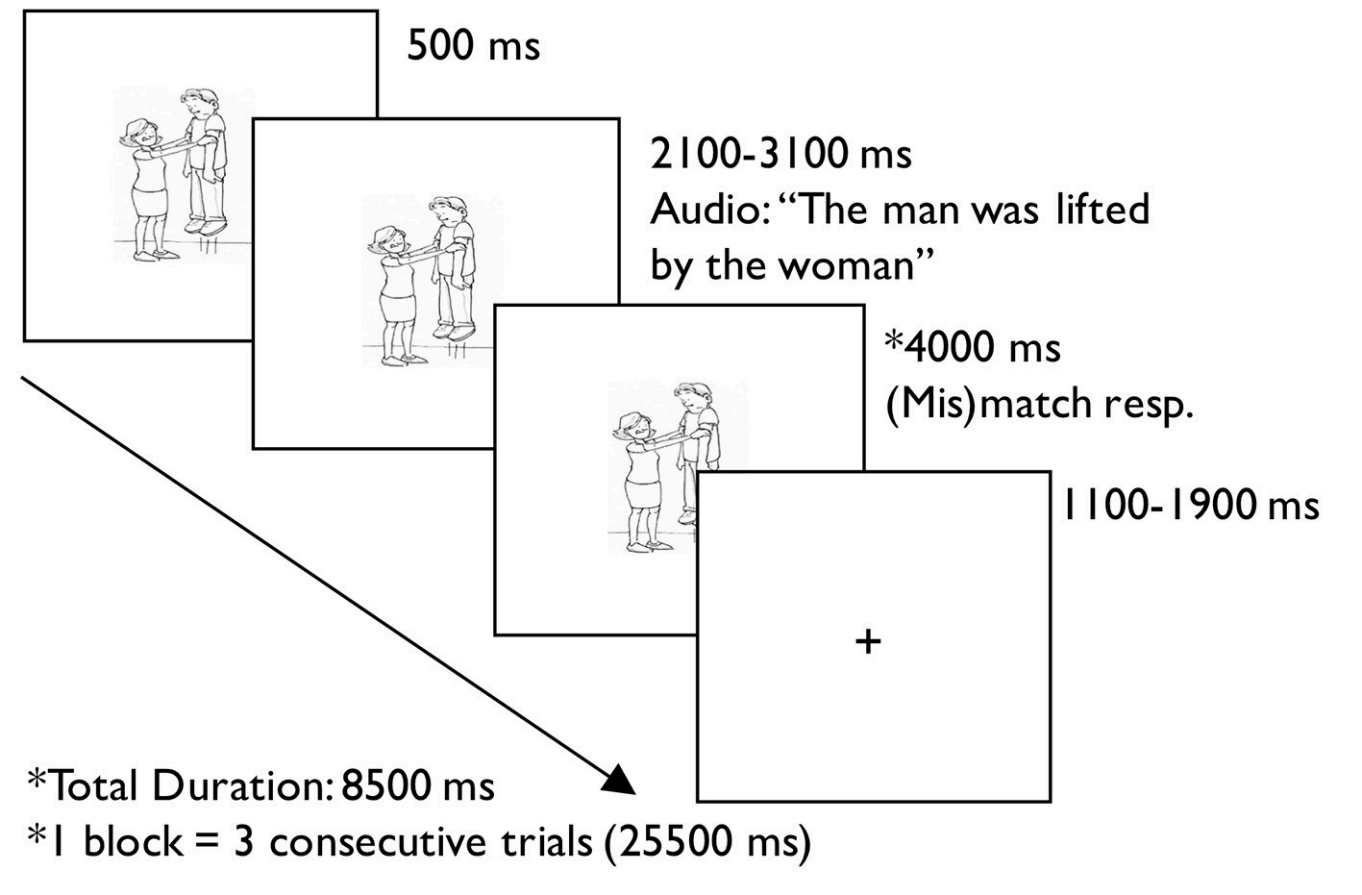
Example trial from the auditory sentence-picture verification task.

Syntactic processing was assessed using an auditory sentence-picture verification task. Before MRI scanning, participants demonstrated understanding of the task and habituation of the scanning environment with practice inside a mock MRI scanner. At the beginning of each trial, participants saw a visual stimulus followed by an auditory stimulus 500 ms later. They decided with a button press whether a picture matched an auditorily presented sentence using a response box in their left hand, one for their index finger and one for their middle finger. For the *sentence* trials, participants pushed the button under their index finger if the sentence matched the picture, and the button under their middle finger if the sentence and the picture were a mismatch. For low-level auditory-visual processing *baseline* trials, participants were instructed to respond with either button after hearing an auditory stimulus. The response period in sentence trials was longer than that in baseline trials because of differences in task difficulty. Each trial ended in a fixation cross of jittered duration due to the varied length of the auditory stimulus, such that the baseline trials were 6.5 s and sentence trials were 8.5 s. The presentation of events for each trial is detailed in Figure 1.

The order of runs for each participant was predetermined using a Latin square design. Since there is evidence that intra-subject reliability is moderate to high across days for language tasks (Wang et al., 2015), some participants completed all four runs on 1day while others completed two runs on day one and two on day two.

### Stimuli

Experimental sentences included 16 frequently occurring, transitive verbs that were semantically reversible, had a regular passive form (-ed), and were easily picturable. The visual stimuli consisted of 32 black and white drawings, two for each verb. In each pair of drawings, one depicted an agent acting upon the theme, and the other reversed the roles of the participants. The auditory stimuli consisted of 128 unique sentences (see Appendix) created by reversing the participant roles in the 16 verbs within four different *sentence* conditions:

1. *Active*: The woman was weighing the boy.
2. *Passive*: The boy was weighed by the woman.
3. *Subject-Cleft (SC)*: It was the woman who weighed the boy.
4. *Object-Cleft (OC)*: It was the boy who the woman weighed.

The first noun phrase in the actives and SCs were agents of the verb and therefore, these sentence types were considered canonical. The first noun phrase in the passives and OCs were not agents of the verb, and therefore, these sentence types were considered non-canonical sentences. The effect of NP-movement can be contrasted by comparing passives and actives as they have a similar syntactic structure. In passives, the moved constituent, *the woman* in (2), occupies an argument position. Similarly, the effect of wh-movement can be contrasted by comparing OCs and SCs because of their similar syntactic structure. Also, both contain the specifier *who* which is linked to agent, *the woman* in (3) and (4). However, in OCs the moved constituent occupies a non-argument position. To control for morphological complexity across conditions, all sentences included past tense verb forms, i.e., past progressive, simple past tense. Given the differences in syntactic structure, cleft sentences were naturally longer than the other two, however, there were no differences in syllable length between the two cleft structures (*M* = 9.0, *SD* = 0.85). A two-sample *t*-test between active (*M* = 7.91, *SD* = 0.87), and passive sentences (*M* = 8.0, *SD* = 0.84) also confirmed no differences in syllable length for these structures [*t*_(93.9)_ = −0.71, *n.s*.]. Auditory recordings were made by a female speaker with typical prosody, normalized to a consistent sound level, and adjusted to a slightly slower than normal speech rate (*M* = 3.36 syllables per second, *SD* = 0.30) using the program Audacity® version 2.0.0^1^.

A low-level auditory-visual processing *baseline* condition was also included. For this condition, 16 time-reversed audio files (four randomly selected from each sentence condition) and 16 baseline visual stimuli were created. Eight of the baseline visual stimuli came from eight randomly selected pictures that were partitioned into 8 × 8 grids and scrambled. The other eight baseline visual stimuli were 180° rotated versions of the eight scrambled pictures.

The experiment included 288 trials: 192 sentences (48 per type) and 96 baseline. Trials were blocked by condition such that there were 3 trials per block, which yielded 64 sentence blocks, and 32 baseline blocks. The trials were pseudorandomized such that the same verb was presented at least one block apart, half of the trials for each sentence condition matched the presented picture and the other half mismatched the picture, and all agents in each sentence block were not all the same gender. The blocks were grouped into four runs, such that runs A and B consisted of *passive, active*, and *baseline* conditions, and runs C and D consisted of *OC, SC*, and *baseline* conditions.

### MRI Data Acquisition

MRI data were acquired using a 32-channel head coil on a 3 Tesla Siemens TRIO system. To obtain an anatomical image of the brain, T1-weighted three-dimensional multi-planar rapid acquisition gradient echo (MPRAGE) sequences recorded 176 slices with a voxel size of 1.0 × 1.0 × 1.0 mm, using a repetition time of 2,300 ms, echo time of 2.91 ms, a flip angle of 9°, and field of view of 256 mm.

During the experimental task, functional MRI blood oxygen level dependent (BOLD) data were acquired such that each image consisted of 41 slices and a voxel size of 1.7 × 1.7 × 3.0 mm. These images were recorded using a repetition time of 2,400 ms, echo time of 20 ms, a flip angle of 90° and a field of view of 220 mm resulting in a matrix size of 129 × 129. All imaging was conducted at the Department of Radiology's Center for Translational Imaging at Northwestern University.

### Behavioral Data Analysis

Accuracy and reaction time (RT) data from the auditory-sentence picture verification task were analyzed using the R software version 3.5.1. With respect to assumptions of normality, non-parametric sign tests of the accuracy data and parametric paired *t*-tests of the RT data from participants were conducted to elucidate any differences between sentence conditions: active vs. passive, OC vs. SC, and canonical vs. non-canonical.

### FMRI Data Analysis

Individual analysis of anatomical and functional neuroimaging data was conducted on the Northwestern University Neuroimaging Data Archive, which allowed for automatic and optimized preprocessing and first-level statistical data analysis pipeline. Anatomical images went through the following preprocessing steps: skull-stripping, segmentation, registration, and normalization. Preprocessing for functional scans began with despiking, censoring data if framewise displacement was 0.5 mm or greater, slice-time correction, co-registration of the anatomical scan to the mean functional volume, regressing signal from white matter and cerebrospinal fluid, normalization of the anatomical and functional scans using the VBM/DARTEL template in Montreal Neurological Institute (MNI) space (2 × 2 × 2 mm resolution), and smoothing using a 6 mm Gaussian kernel. Scripts from Analysis of Functional NeuroImages (AFNI), FMRIB Software Library (FSL), and Statistical Parametric Mapping 8 (SPM8) were utilized for preprocessing. More details about this implementation can be found in Alpert et al. (2016).

In the first-level statistical analysis, a high pass filter of 128 s was used to eliminate scanner drift. A general linear model containing passive, active, OC, SC, and baseline conditions was specified and estimated in SPM8. Activation for general sentence processing was found with the contrast (*Passive* + *Active* + *OC* + *SC*) > *Baseline*, alternatively referred to as *Sentences* > *Baseline*. Activation for non-canonical compared to canonical sentence processing was identified with the contrast (*Passive* + *OC*) > (*Active* + *SC*), alternatively referred to as *Noncanonical* > *Canonical*. Preferential activation for processing wh-movement was defined by the (*OC* > *SC)* > (*Passive* > *Active*) contrast, or *Wh* > *NP-movement*, whereas (*Passive* > *Active*) > (*OC* > *SC*) was used for isolating activation associated with NP-movement, or *NP* > *Wh-movement*.

At the group level, regions involved with all sentences (both canonical and non-canonical), non-canonical sentence processing, and wh- vs. NP-movement were identified. For each contrast of interest, a one-sample *t*-test of the group's images was conducted with age as a covariate in SPM8. This statistical analysis yielded a binary mask of active voxels common to all participants and an image of the voxel-wise variance of error from the *t*-test. The binary mask and variance of error image were input into 3dFWHMx program from Analysis of Functional NeuroImage (AFNI) to estimate noise smoothness in x-, y-, and z-directions. This was done by fitting the data to a Gaussian plus mono-exponential mixed model because functional MRI data do not have a Gaussian-shaped autocorrelation function, as previously assumed (Eklund et al., 2016). To determine the maximum size of false positive (noise-only) clusters, the estimated noise smoothness, binary mask, and image of variance of error were used in AFNI's 3dClustSim to calculate cluster-defining thresholds at α = 0.01 level of significance and a specified voxel-wise threshold (*p* < 0.001, uncorrected). This program used Monte-Carlo simulations given a specified voxel-wise level of significance. The cluster-defining threshold was reported using 2-sided thresholding since we were interested in both directions of the contrasts of interest, and with first-nearest neighbor clustering, because it produces the most conservative result. The Harvard-Oxford atlas was used to throughout the study to label peak activation.

### Effective Connectivity Analysis

Effective connectivity describes how neural activity from one region influences neural activity of another region. Dynamic Causal Modeling (DCM) is a hypothesis-driven method for estimating effective connectivity using functional MRI data (Friston et al., 2003). DCM is particularly useful for testing hypotheses about the influences of particular connections within a neural network, e.g., how sensory stimuli or experimental tasks modulate neuronal interactions. The method is dynamic because it uses differential equations to estimate connectivity and task-induced neuronal interactions, and causal because directionality can be specified (Seghier et al., 2010; Stephan et al., 2010). For task-based functional MRI studies, experimental conditions serve as input into the model by either driving neural activity throughout the network and/or modulating connectivity between regions.

The first step in DCM is selecting the nodes in the network and identifying connections. For this study, node selection and connectivity were guided by neurocognitive models of sentence comprehension. The DCM12 toolbox in SPM12 was used for effective connectivity analysis. Each model was specified using binary values in three matrices: the A-matrix, B-matrix, and C-matrix. The A-matrix is an *n*-by-*n* square matrix representing the intrinsic connectivity (i.e., in the absence of external input) between the *n* nodes. The B-matrix is an *n*-by-*n*-by-*c* matrix representing how *c* experimental conditions causes a change in the rate of neural activity between the *n* nodes. The C-matrix is an *n*-by-*i* matrix representing the *i* external inputs that would affect the rate of change of neural activity of *n* nodes which consequently drives activity within the model, e.g., the “driving input.” This matrix triplet, signifying one model, and the fMRI data were inputs to the DCM12 toolbox to estimate model parameters and calculate model fit using Bayesian statistics.

#### Node Selection

For the present study, nodes in the network were specified from group peaks identified in the *Noncanonical* > *Canonical* contrast (*p* < 0.001, uncorrected; *k* > 25) masked by the *All Sentences* > *Baseline* contrast (*p* < 0.001, uncorrected) to isolate sentence processing regions involved in processing both wh- and NP-movement. One of the peak coordinates (−48, 22, 22) did not have a label in the Harvard-Oxford Atlas, but it was labeled as left pars opercularis of the inferior frontal gyrus (LIFGop) because the cluster extended primarily into that region. This resulted in peaks within the LIFGop, left medial superior frontal gyrus (LSFGm), and left middle frontal gyrus (LMFG), and left posterior superior temporal gyrus (LSTGp). The same contrast and mask were applied to all first-level analyses to identify suprathreshold voxels (*p* < 0.05, uncorrected) within a 12 mm radius sphere centered at each of the group peaks (or local maxima if <10 suprathreshold voxels were yielded using the group peak). Subject-specific eigenvariates were adjusted for effects-of-interest and extracted from a modified general linear model using these suprathreshold voxels as a mask. Regions were excluded from the DCM analysis if consistent activation was not observed across subjects. In addition, subjects were excluded from the DCM analysis if suprathreshold voxel-wise activation was <10 voxels for at least one of the resulting nodes. These exclusionary criteria were imposed to decrease the likelihood of incorporating noisy data during model estimation. The modified general linear model concatenated all 4 runs, modeled the 5 conditions, and regressed for all 4 runs and linear drift for each run.

#### Model Specification and Estimation

In the present study, neuronal connections within models were assumed to be bilinear and deterministic (see Seghier et al., 2010 for a description of all model specification options) which are appropriate for neurologically normal participants. Further, two-state neuronal equations were used to improve model estimation by quantifying the interaction between inhibitory and excitatory neuronal subpopulations within a given region (Marreiros et al., 2008). Unlike modeling with one-state neuronal equations, positive constraints (or priors) for between-region connections and negative constraints on within-region connections were implemented for determining intrinsic connectivity (e.g., A-Matrix). Two-state DCM also estimated the proportional increase or decrease from intrinsic connectivity between regions caused by task-induced perturbations (e.g., B-Matrix). Parameters for intrinsic connectivity and modulations were log scaled. For statistical analysis, they were exponentially transformed such that a value of 1 represents no neural rate of change from region X to region Y; a value <1 represents a decrease in neural rate of change from region X to region Y; and values >1 represent an increase in the neural rate of change from region X to region Y. Parameters for external driving input (e.g., C-Matrix) are estimated in hertz.

**Figure 2:**
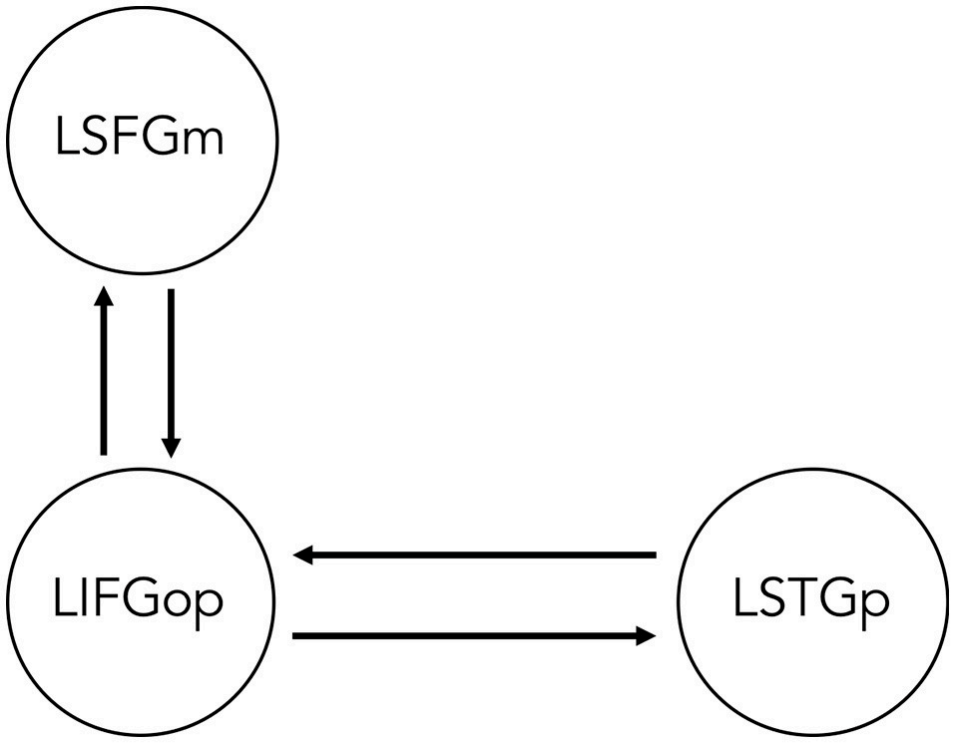
Base dynamic causal model of network of regions involved in non-canonical sentence processing for sentence-picture verification task. LSFGm, Left superior frontal gyrus, medial part. LIFGop, left inferior frontal gyrus, opercular part. LSTGp, Left superior temporal gyrus, posterior part.

Two sets of models for each movement type were specified and estimated, such that *All Sentences* > *Baseline* contrast was indicated as the driving input (i.e., sentence processing driving neural activity within the network) and either the *OC* > *SC* contrast (i.e., processing wh-movement) or the *Passive* > *Active* contrast (i.e., processing NP-movement) modulated connectivity between regions. Specifying all three contrasts within the same GLM would leave no variance for the model. Therefore, it was not possible to directly compare models of wh- and NP-movement in a statistical way. Figure 2 illustrates the bidirectional intrinsic connections specified between the LIFGop and LSTGp, as they are connected by the superior longitudinal fasciculus, and between the LIFGop and LSFGm, by way of the frontal aslant tract (Catani et al., 2013; Dick et al., 2014; Martino and Lucas, 2014).

To create different plausible models, driving input either entered the LIFGop, LSTGp, or both the LIFGop and LSTGp. In addition, syntactic movement either modulated the connection from the LIFGop to the LSTGp (LIFGop-LSTGp), LSTGp-LIFGop, or both connections. Syntactic movement was also modeled such that it either did not modulate connectivity between the LIFGop and LSFGm, it only modulated LIFGop-LSFGm, or it modulated both LIFGop-LSFGm and LSFGm-LIFGop. Models were excluded if connectivity to, but not from, the driving input region was modulated by syntactic movement, as this overemphasizes the neural activity in the driving input region. This resulted in two sets of 21 models (see Table 1 for details). Each set contained models that estimated the effect of wh-movement or NP-movement on connectivity. Model evidence, the probability of observing the fMRI data given the model's specifications, was calculated for every single model.

**Table 1:** Full model space for DCM analysis (1 = included, 0 = not included).

	**Driving input**		**Modulated by syntactic movement**			
**Model ID**	**IFGop**	**STGp**	**IFG-STG**	**STG-IFG**	**IFG-mSFG**	**mSFG-IFG**
M1	1	1	1	1	1	1
M2	1	1	1	1	1	0
M3	1	1	1	1	0	0
M4	1	1	1	0	1	1
M5	1	1	0	1	1	1
M6	1	1	1	0	1	0
M7	1	1	0	1	1	0
M8	1	1	1	0	0	0
M9	1	1	0	1	0	0
M10	1	0	1	1	1	1
M11	1	0	1	1	1	0
M12	1	0	1	1	0	0
M13	1	0	1	0	1	1
M14	1	0	1	0	1	0
M15	1	0	1	0	0	0
M16	0	1	1	1	1	1
M17	0	1	1	1	1	0
M18	0	1	1	1	0	0
M19	0	1	0	1	1	1
M20	0	1	0	1	1	0
M21	0	1	0	1	0	0

#### Bayesian Model Selection and Averaging

For each type of syntactic movement, the 21 models were grouped into three different families: those with driving input into the LIFGop, into the LSTGp, or into both. A random-effects family-wise Bayesian Model Selection (BMS) was performed and the winning family was that which had the highest exceedance probability. The exceedance probability is the likelihood that a particular family of models, compared to the other families, generated the data of a randomly selected participant from the group. Therefore, the sum of the exceedance probabilities of all families in the BMS equaled 1. The winning family was subject to Bayesian Model Averaging (BMA) to obtain a model average which contained parameters weighted by the posterior probability of each contributing model within the family. Subject-specific parameters from the A-, B-, and C-matrices were entered into one-sample *t*-tests and corrected for multiple comparisons via false discovery rate (FDR) to determine whether the estimated intrinsic connectivity was significantly different from the prior constraint, whether the estimated modulatory effect was significantly different from no effect, and whether the estimated effect of the driving input was significantly >0 Hz. To determine whether particular connections were significantly stronger than others, all intrinsic connections and modulated connections were entered into separate general linear models in order to conduct simultaneous pair-wise comparisons.

## Results

### Behavioral Results

A significant canonicity effect in accuracy was found such that participants were more accurate across the two canonical sentence types (*median* = 1) vs. the two non-canonical sentence types (*median* = 0.98), *p* < 0.005. This effect was primarily driven by higher accuracy for SC sentences (*median* = 0.99) compared to OC sentences (*median* = 0.98), *p* = 0.06. No statistically significant difference in accuracy was observed between passives and actives. With the exception of one participant whose accuracy ranged from 83 to 94%, all other participants were 90% accurate or greater across all 4 structures. Participants were also significantly quicker to respond [*t*_(20)_ = 8.55, *p* < 0.005] to both canonical sentences types (*M* = 2776 ms, *SD* = 2 45) compared to the two non-canonical sentences types (*M* = 2,934, *SD* = 282). They had a significantly faster RT for actives (*M* = 2,642, *SD* = 247) compared to passives [*M* = 2,759, *SD* = 289; *t*_(20)_ = 5.36, *p* < 0.005] and for SCs (*M* = 2,903, *SD* = 260) compared to OCs [*M* = 3,092, *SD* = 315; *t*_(20)_ = 5.76, *p* < 0.005]. Direct comparison of the non-canonical structures showed no difference in accuracy [*t*_(20)_ = 0.37, *p* = 0.71], but a significantly faster reaction time for passives compared to OCs [*t*_(20)_ = 5.91, *p* < 0.001].

### FMRI Results

**Figure 3:**
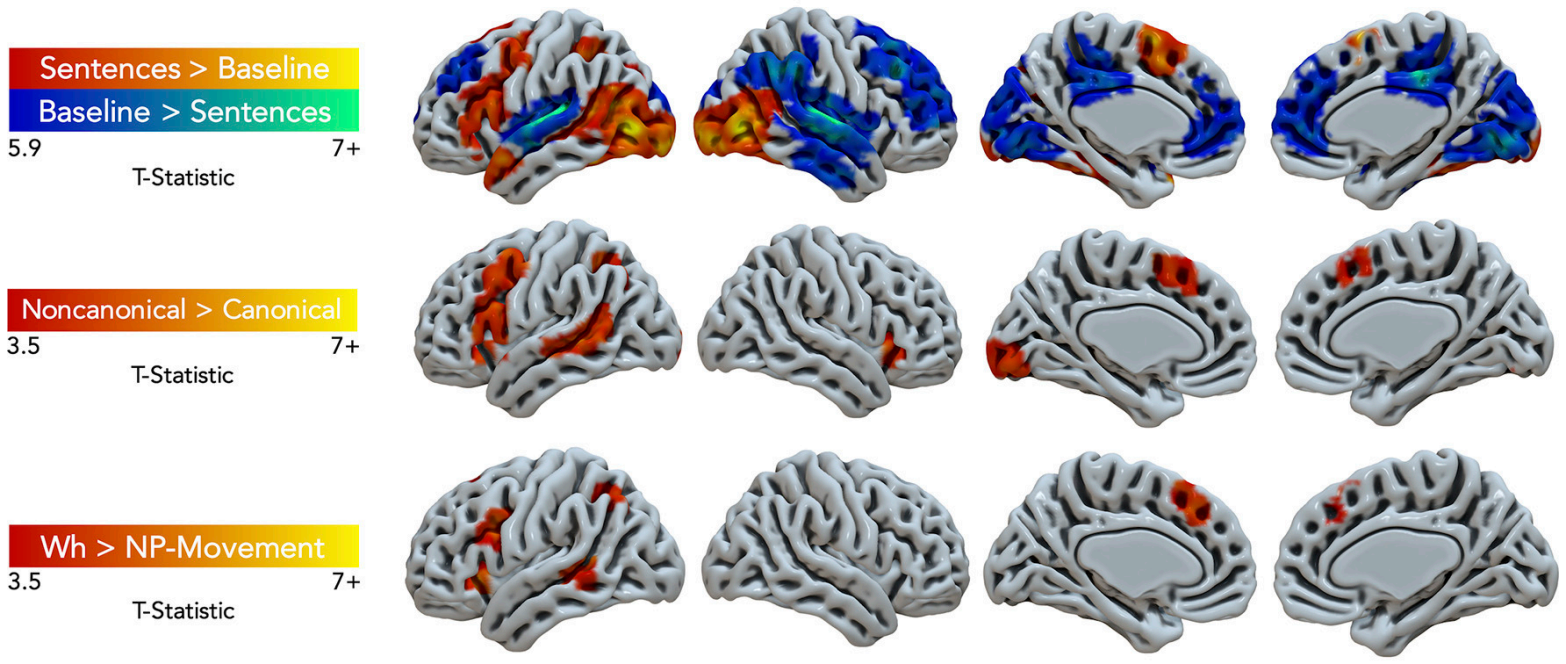
Significant fMRI activation (uncorrected voxelwise *p* < 0.001) of the contrasts *Sentences* > *Baseline* and vice-versa (corrected cluster-defining threshold *k* > 61), *Non-canonical* > *Canonical* (*k* > 43.4), and *Wh* > *NP-movement* (*k* > 42.1) from healthy adult participants.

Task performance elicited large clusters of activation primarily in the left hemisphere for general sentence processing (*All Sentences* > *Baseline*) (cluster-defining threshold was *k* = 61; Figure 3, top row, red-yellow gradient). Peak activations were in the left pars triangularis of the IFG, middle frontal gyrus (MFG), supplementary motor area (SMA), temporal pole, SPL, superior LOC (LOCs), occipital pole, cerebral white matter, and right inferior lateral occipital cortex (LOCi) (Table 2). The Harvard-Oxford atlas did not have a label for the peak located at (10, −68, −24), but it appeared to be located within the right cerebellum. The opposite contrast, activation for the baseline condition compared to the all sentence conditions, yielded peak activation in the bilateral paracingulate gyrus, SFG, right planum temporale, lingual gyrus, posterior supramarginal gyrus (SMGp), MTGp, left planum polare, and posterior cingulate gyrus (Figure 3, top row, blue-green gradient). No significant regions of activation were elicited for canonical compared to non-canonical sentences and for NP- compared to wh-movement.

**Table 2:** Summary of peak activation in MNI space.

**Contrast**	**L/R**	**Peak location**	**k**	**T**	**x**	**y**	**z**
Sentences > baseline	L	IFGtri	480	6.2	−50	28	18
	R	SMA	217	9.0	−4	8	60
	L	Temporal pole	127	7.7	−54	8	−18
	L	Cerebral white matter	112	6.3	−14	−2	14
	L	MFG	432	6.7	−30	−4	56
	L	Superior parietal lobule (SPL)	190	7.3	−28	−46	44
	R	N/A	104	9.2	10	−68	−24
	L	LOCs	73	6.5	−26	−70	32
	R	LOCi	2,285	11.2	48	−76	8
	L	Occipital pole	3,271	11.3	−26	−100	−8
Baseline > sentences	R	Paracingulate gyrus	462	−6.9	6	50	4
	R	Paracingulate gyrus	131	−6.3	4	42	28
	L	SFG	429	−6.9	−20	28	40
	R	SFG	1,844	−8.8	20	26	56
	L	Planum polare	1,910	−13.9	−48	−10	−2
	R	Planum temporale	2,347	−10.8	60	−12	6
	R	MTGp	146	−6.9	58	−12	−30
	R	Posterior cingulate gyrus (pCG)	730	−9.3	2	−28	32
	R	SMGp	941	−10.3	56	−40	40
	R	Lingual gyrus	2,069	−9.7	18	−74	−4
Non-canon > canonical	R	Frontal orbital cortex	63	5.1	38	22	−2
	L	IFGop	271	6.8	−48	22	22
	L	MFG	132	6.2	−50	16	38
	L	Paracingulate gyrus	67	5.4	−2	14	52
	L	MTGp	308	10.5	−52	−36	0
	L	LOCs	50	6.0	−34	−62	46
	R	N/A	44	6.0	10	−74	−24
	L	Occipital fusiform gyrus (OFG)	120	5.6	−14	−86	−12
Canonical > Non-canon		No significant clusters of activation					
Wh > NP-Movement	L	SFGm	47	5.7	−6	30	46
	L	Insular cortex	70	8.7	−32	24	−2
	L	IFGop	167	7.6	−36	16	24
	L	MTGp	49	5.3	−56	−42	2
	L	LOCs	68	6.3	−36	−58	46
NP > Wh-Movement		No significant clusters of activation					

Non-canonical sentence processing elicited peak activity (cluster-defining threshold was *k* = 43.4; Figure 3, middle row, red-yellow gradient) in the left hemisphere. This included the left pars opercularis of the IFG, MFG, paracingulate gyrus, MTGp, LOCs, and occipital fusiform gyrus (Table 2).

Contrasting wh- compared to NP-movement structures (cluster-defining threshold was *k* = 42.1; Figure 3, bottom row, red-yellow gradient) also showed a left hemisphere network of perisylvian regions. Wh-movement elicited peak activity in the medial SFG (SFGm), insular cortex, pars opercularis of the IFG, MTGp, and LOCs (Table 2).

### Effective Connectivity Results

**Figure 4:**
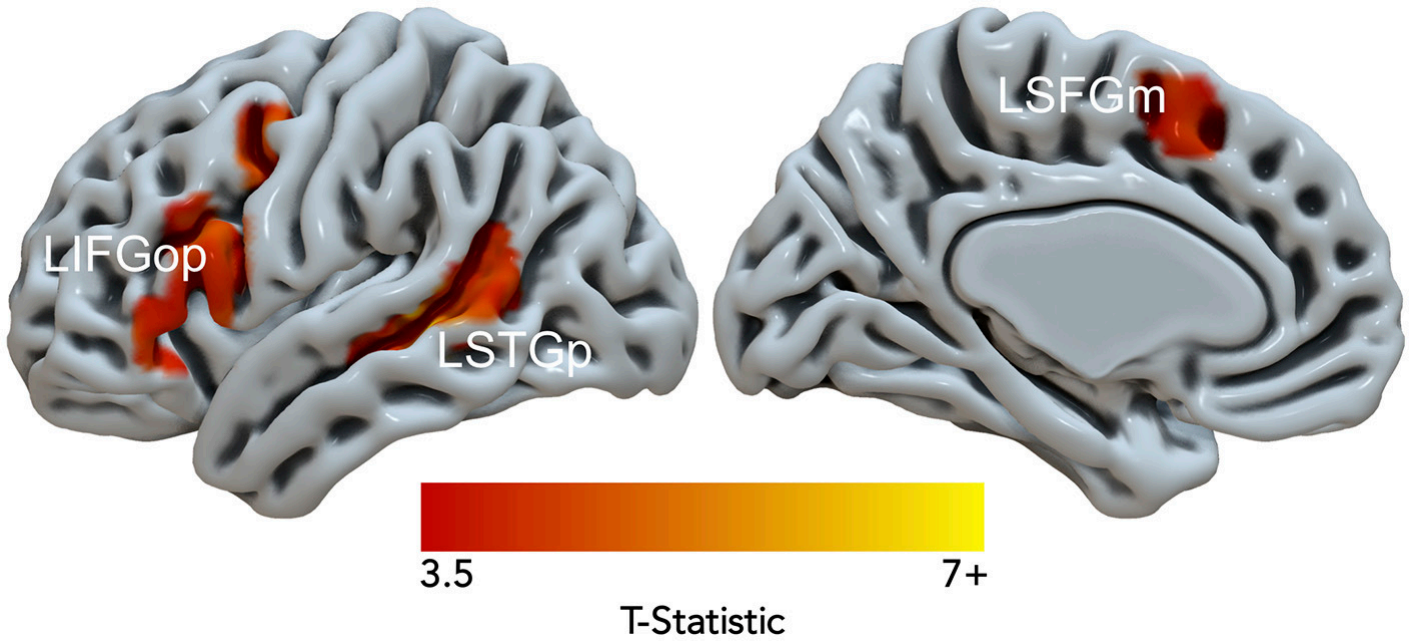
Significant fMRI activation (uncorrected voxelwise *p* < 0.001) of *Non-canonical* > *Canonical* (corrected cluster-defining threshold *k* > 61) masked by *Sentences* > *Baseline* (*k* > 61) from healthy adult participants.

Data from 15 of the 21 participants were used in the DCM analysis (7 females; age in years: *M* = 33.6, *SD* = 10.8; education in years: *M* = 17.9, *SD* = 2.5). Six subjects were excluded because they had < 10 suprathreshold voxels for at least one of three resulting nodes. Figure 4. illustrates the *Noncanonical* > *Canonical* contrast (*p* < 0.001, uncorrected; *k* > 25) masked by the *Sentences* > *Baseline* contrast (*p* < 0.001, uncorrected). Table 3 reports the peak activation in MNI space which included peaks within the LIFGop, left posterior superior temporal gyrus (LSTGp), left medial superior frontal gyrus (LSFGm), and left middle frontal gyrus (LMFG). First-level analyses revealed inconsistent activation within the MFG across subjects demonstrating that this region's neural response was driven by a subset of participants. Therefore, the MFG was not included as a node in the DCM analysis.

**Table 3:** Peak and sub-peak activation for Non-canonical > canonical masked by sentences > baseline.

**L/R**	**Location of peaks and submaxima peaks (indented)**	**k**	**T**	**x**	**y**	**z**
L	Inferior frontal gyrus, opercular part (LIFGop)	121	6.78	−48	22	22
	- Inferior frontal gyrus, opercular part		5.24	−54	16	18
	- Inferior frontal gyrus, triangular part		5.04	−52	20	−2
L	(Medial) Superior frontal gyrus (LSFGm)	28	5.19	−6	16	50
	- (Medial) Superior frontal gyrus		4.46	−4	10	58
L	Middle frontal gyrus (LMFG)	33	5.10	−42	2	48
L	Superior temporal gyrus, posterior division (LSTGp)	148	10.48	−52	−36	0
	- Angular gyrus		5.54	−54	−52	10
	- Middle temporal gyrus, temporo–occipital part		5.24	−48	−48	6

#### Wh-Movement Models

**Figure 5:**
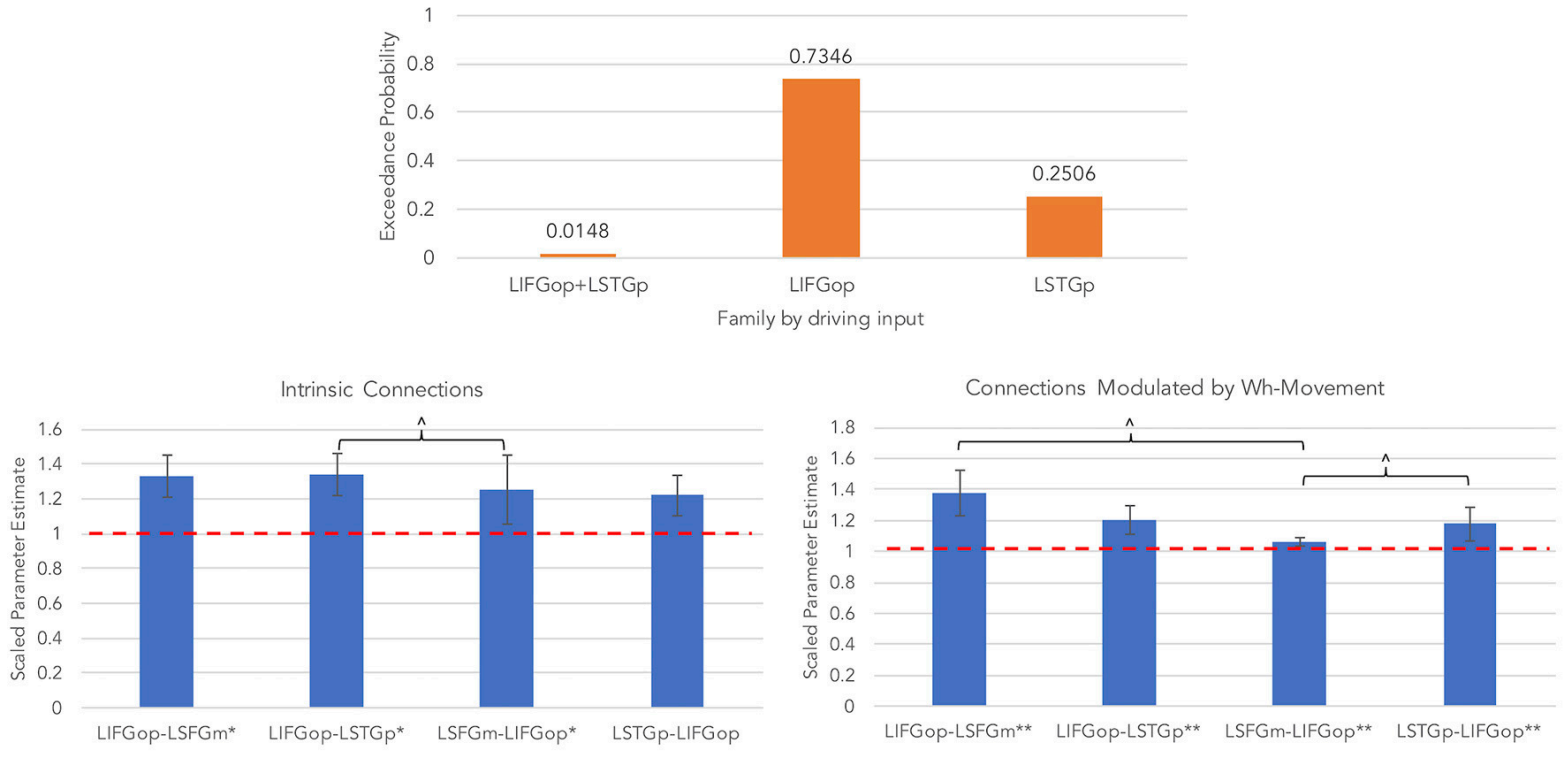
Results for wh-movement models (^**^p < 0.05, FDR-corrected; ^*^p < 0.05, uncorrected; ^∧^*p* < 0.08, uncorrected).

An initial random-effects BMS was conducted among the 21 models and there was no clear winning model (highest exceedance probability = 0.38, next highest exceedance probability = 0.20). Provided these results, a random-effects family-wise BMS was conducted and is illustrated in Figure 5 *(top panel)*. Among the 3 model families, the winning family was the set of models with driving input into the LIFGop (exceedance probability = 0.73) with the next best winning family being the set of models with driving input into the LSTGp (exceedance probability = 0.25).

BMA was conducted across the 6 models with input into the LIFGop (models 10–15, see Table 1) yielding averaged model parameters weighted by their posterior probability. Inspection of individual data resulted in exclusion of one participant because their estimated parameters were >3 standard deviations from the mean. Figure 5 (bottom left panel) displays the parameters for intrinsic connections in which the red-dotted line, equal to the value of 1, denotes no estimated difference from the prior. Estimated parameters for intrinsic connections were greater than the prior for the LIFGop-LSFGm (*M* = 1.24, *SD* = 0.33; *p* < 0.05, uncorrected), LIFGop-STGp (*M* = 1.27, *SD* = 0.40; *p* < 0.05, uncorrected), LSFGm-LIFGop (*M* = 1.06, *SD* = 0.10; *p* < 0.05, uncorrected), and LSTGp-LIFGop (*M* = 1.13, *SD* = 0.25; *n.s*.). Simultaneous pairwise comparisons of intrinsic connections revealed a trend toward significance (*p* < 0.08, uncorrected) between the LIFGop-STGp and LSFGm-LIFGop [*t*_(3, 52)_ = −1.87]. Driving input into the LIFGop was significantly >0 (*M* = 0.03 Hz, *SD* = 0.02, *p* < 0.001).

Figure 5 (bottom right panel) displays the parameters for connections modulated by wh-movement in which the red-dotted line, equal to the value of 1, denotes no estimated difference from the intrinsic connection when processing wh-movement from region X to region Y. Wh-movement significantly modulated all connections after a FDR correction for multiple comparisons, *p* < 0.05: LIFGop-LSFGm (*M* = 1.24, *SD* = 0.27), LIFGop-LSTGp (*M* = 1.22, *SD* = 0.38), LSFGm-LIFGop (*M* = 1.04, *SD* = 0.05), and LSTGp-LIFGop (*M* = 1.21, *SD* = 0.28). Simultaneous pairwise comparisons of modulatory connections also revealed a trend toward significance (*p* < 0.08, uncorrected) between the LIFGop-LSFGm and LSFGm-LIFGop, *t*_(3, 52)_ = −2.00, and between the LIFGop-STGp and LSFGm-LIFGop, *t*_(3, 52)_ = −1.79. Table 4 summarizes the statistical analyses of the mean parameter estimates from the BMA models modulated by wh-movement in which a value of 1 denotes no estimated perturbation in neural rate of change intrinsically or in response to processing wh-movement from one region to the other.

**Table 4:** Mean (and standard deviation) of subject-specific scaled BMA parameters for wh-movement models.

**Connection**	**Intrinsic connectivity**	**Modulation by Wh-Mov**
IFGop-SFGm	^*^1.24 (0.33)	^**^1.23 (0.27)
IFGop-STGp	^*^1.27 (0.40)	^**^1.22 (0.38)
SFGm-IFGop	^*^1.06 (0.10)	^**^1.04 (0.05)
STGp-IFGop	1.13 (0.25)	^**^1.21 (0.08)

** p < 0.05, FDR-corrected;

* *p < 0.05, uncorrected*.

#### NP-Movement Models

**Figure 6:**
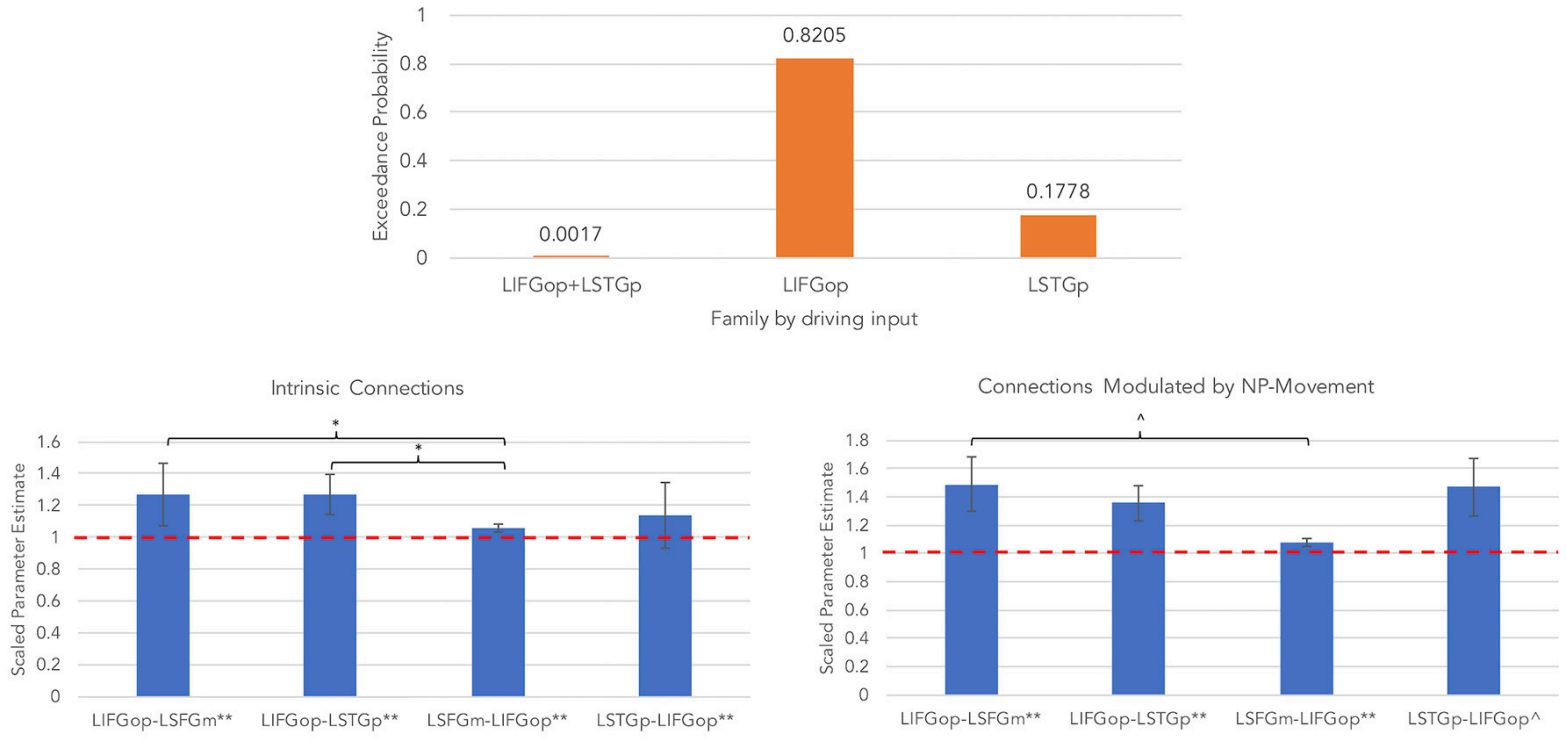
Results for NP-movement models (^**^p < 0.05, FDR-corrected; ^*^p < 0.05, uncorrected; ^∧^*p* < 0.08, uncorrected).

An initial random-effects BMS was conducted among the 21 models and there was also no clear winning model for the NP-movement models (highest exceedance probability = 0.46; next highest exceedance probability = 0.29). Provided these results, a random-effects family-wise BMS was conducted and is illustrated in Figure 6 (top panel). Among the 3 model families, the winning family was the set of models with driving input into the LIFGop (exceedance probability = 0.82) with the next best winning family being the set of models with driving input into the LSTGp (exceedance probability = 0.18).

Similar to the wh-movement results, BMA was conducted across the 6 models with input into the LIFGop for NP-movement (models 10–15, see Table 1). Figure 6 (bottom left panel) displays the parameters for intrinsic connections in which the red-dotted line, equal to the value of 1, denotes no estimated difference from the prior. Parameters for intrinsic connections were greater than the prior for all connections, *p*(FDR) < 0.05: LIFGop-LSFGm (*M* = 1.27, *SD* = 0.31), LIFGop-LSTGp (*M* = 1.27, *SD* = 0.32), LSFGm-LIFGop (*M* = 1.06, *SD* = 0.07), and LSTGp-LIFGop (*M* = 1.14, *SD* = 0.21). Simultaneous pairwise comparisons of intrinsic connections revealed a difference (*p* < 0.05, uncorrected) between the LIFGop-LSFGm and LSFGm-LIFGop, *t*_(3, 56)_ = −2.38, and between the LIFGop-LSTGp and LSFGm-LIFGop, *t*_(3, 56)_ = −2.34. Driving input into the LIFGop was significantly >0 (*M* = 0.03 Hz, *SD* = 0.02; *p* < 0.001). Figure 6 (bottom right panel) displays the parameters for connections modulated by NP-movement in which the red-dotted line, equal to the value of 1, denotes no estimated difference from the intrinsic connection when processing NP-movement from region X to region Y. NP-movement modulated LIFGop-LSFGm (*M* = 1.49, *SD* = 0.75; *p*(FDR) < 0.05), LIFGop-STGp (*M* = 1.36, *SD* = 0.50; *p*(FDR) < 0.05), LSFGm-LIFGop (*M* = 1.07, *SD* = 0.10; *p*(FDR) < 0.05), and LSTGp-LIFGop (*M* = 1.21, *SD* = 0.28; *p* < 0.05, uncorrected). Simultaneous pairwise comparisons revealed a difference between the LIFGop-LSFGm and LSFGm-LIFGop (*t*_(3, 56)_ = −1.91; *p* = 0.06, uncorrected). Table 5 summarizes the statistical analyses of the mean parameter estimates from the BMA models modulated by NP-movement in which a value of 1 denotes no estimated perturbation in neural rate of change intrinsically or in response to processing NP-movement from one region to the other.

**Table 5:** Mean (and standard deviation) of subject-specific BMA parameters for NP-movement models.

**Connection**	**Intrinsic connectivity**	**Modulation by NP-Mov**
IFGop-SFGm	^**^1.27 (0.31)	^**^1.49 (0.75)
IFGop-STGp	^**^1.27 (0.32)	^**^1.36 (0.50)
SFGm-IFGop	^**^1.06 (0.07)	^**^1.07 (0.10)
STGp-IFGop	^**^1.14 (0.21)	^∧^1.44 (0.80)

** p < 0.05, FDR-corrected; ^*^ p < 0.05, uncorrected; ^∧^p < 0.08, uncorrected.

## Discussion

The primary aims of this study were to identify the neural network associated with the comprehension of complex sentences and to explore how syntactic complexity modulated connectivity within this network. Using an auditory sentence-picture verification fMRI task, this study demonstrated that non-canonical sentences with wh-movement elicit greater neural activity than those with NP-movement, though both types of movement modulate neural connectivity in a similar manner. While findings from the fMRI analysis support the idea that processing the wh-movement operation requires more neurocognitive resources than the NP-movement, results from the connectivity study suggest that both movement operations may undergo the same stages of processing.

First, results from the fMRI analysis revealed that all sentence conditions compared to the baseline condition (*All Sentences* > *Baseline*) yielded a mostly left hemisphere network with peaks in the left pars triangularis of the IFG, right SMA, left temporal pole, MFG, SPL, LOCs, right LOCi, and left occipital pole. As many of these regions have been previously found in neuroimaging studies of sentence processing (see, Friederici, 2011 for a comprehension review), these results are validating. Bilateral occipital activation was observed in visual association cortex which responds more to complex visual representations than simple visual stimuli (Van Essen and Maunsell, 1983). The opposite contrast (*Baseline* > *All Sentences*) resulted in a bilateral network of regions with peaks in bilateral paracingulate gyrus, SFG, left planum polare, right planum temporale, MTGp, posterior cingulate gyrus, SMGp, and lingual gyrus. Activation in bilateral STG fell within the primary auditory cortex and likely reflected the contrast between hearing reversed speech vs. spoken sentences (Skipper, 2014). The other medial and right hemisphere regions were previously reported when comparing less to more cognitively demanding tasks (Raichle and Snyder, 2007). Therefore, they may reflect differences in cognitive functioning or effort between *baseline* and *sentence* conditions.

In line with previous reports of noncanonical sentence processing (Bornkessel et al., 2005; Caplan et al., 2008; Thompson et al., 2010b; Bornkessel-Schlesewsky et al., 2012; Makuuchi et al., 2012; Mack et al., 2013), this experiment yielded a mostly left hemisphere network with peaks in pars opercularis of the IFG, MFG, paracingulate gyrus, MTGp, and LOCs during non-canonical sentence comprehension when compared to canonical sentences (*Noncanonical* > *Canonical*), while no significant activation was found for the opposite contrast (*Canonical* > *Noncanonical*). Non-canonical sentence processing, examined by combining the wh- and NP-movement contrasts (e.g., *OC* + *Passive* > *SC* + *Active*), requires forming a dependency between the moved constituent and the trace site resulting in reactivation of the filler after the verb is encountered to initiate re-assignment of its thematic role. We found that the left IFG and MTGp were two regions active for this contrast, which is consistent with both models of auditory sentence comprehension tested (Friederici, 2012; Bornkessel-Schlesewsky and Schlesewsky, 2013). Both predict involvement of these regions in processing non-canonical sentences, but disagree with regard to their function. According to the model by Bornkessel-Schlesewsky and Schlesewsky (2013), lexical-semantic processing first takes place in left posterior temporal regions followed by combinatorial syntactic and thematic processing in dorsal and ventral pathways, respectively, to the left IFG where these two types of information are integrated. On the other hand, Friederici (2012) claims that the left IFG is involved with assigning grammatical relations between syntactic constituents, which precedes involvement of the left posterior superior temporal cortex in re-assigning thematic roles.

Our results also showed that wh-movement elicits greater activity in left inferior frontal and posterior temporal cortices compared to NP-movement which may reflect greater processing resources for thematic role assignment in the context of the double-dependency seen in wh-movement. This novel finding (*Wh* > *NP-movement*) revealed a left perisylvian network with peak activity in the SFGm, insular cortex, pars opercularis of the IFG, MTGp, and LOCs, but no significant activity vice-versa, which provides support for both representational and processing accounts of wh-movement. Representationally, object-cleft sentences entail movement across clausal boundaries, i.e., a type of A-bar movement in which the moved constituent, *who*, occupies a non-argument position, the specifier position of the Complement Phrase. This results not only in a co-referential relation between the moved constituent (*who*) and the trace (as in NP-movement structures), but also between *who* and the head noun of the matrix clause. In contrast, NP-movement is a type of A-movement and it occurs when the displaced constituent, the filler, occupies an argument position and leaves behind a trace. In the passive sentences used in the study, the filler occupies the subject position in the syntactic frame because it is an argument of the verb. The trace forms a direct dependent (co-referential) relationship with the noun phrase. Although both object-cleft sentences and passives were highly accurate, reaction times were longer for the wh-movement structures compared to the NP-structures which provides additional evidence for processing differences. Wh-movement elicited activity in the left pars opercularis of the IFG and the left insular cortex, consistent with the model of auditory sentence comprehension by Friederici (2012) describing the left pars opercularis' role in processing higher-order syntactic relations.

In addition, *Wh* > *NP-movement* elicited activity in a subset of the regions observed in the *Noncanonical* > *Canonical* contrast, namely the SFGm and LOCs, which may reflect processes shared between the two movement types, but require additional computational resources for wh-movement. From a linguistic standpoint, the left frontal activation has been reported for effortful sentence comprehension (Adank, 2012a,b) as well as word sequencing (Crozier et al., 1999; Alario et al., 2006) which is more relevant for OC sentences due to the non-canonical word order. The peak within the LOCs is rostrally adjacent to both the SPL and the angular gyrus. Activation in this area, particularly the angular gyrus, has been associated with processing thematic relations between words (Kalénine et al., 2009; Boylan et al., 2015, 2017; Lewis et al., 2015). Thompson and Meltzer-Asscher (2014) argues that the function of the AG is the retrieval or argument structure information within their model of verb argument structure processing. However, some claim that these regions instead play a domain-general role in language processing because they fall outside of the more conventional left frontotemporal syntax processing network (see Campbell and Tyler (2018) for more details on this argument). The left frontal activation in this study is located dorsally to the inferior frontal gyrus, putatively involved in syntactic processing, and has been associated with the domain-general multiple demand network (Campbell et al., 2016). In addition, the left LOCs and the area rostrally adjacent have been previously linked to attentional processing (Dreher and Grafman, 2003; Mizuno et al., 2012). Further investigation is required to determine whether these regions are specifically relevant for syntactic processing or support language processing in general.

The effective connectivity analysis demonstrated that syntactic movement modulated both temporofrontal and frontotemporal pathways, where external input to the LIFGop drove neural activity throughout the network. Two related studies previously examined how syntactic complexity modulated connectivity between language regions. den Ouden et al. (2012) used a similar auditory sentence-picture verification task to examine how syntactic complexity modulated the network. Their result was a model in which the LIFG's response to sentences drove network activity, and OC sentences modulated the connection from the LIFG to the LSTGp. Similarly, Makuuchi and Friederici (2013) employed a sentence verification task to determine how complex sentences modulated activity in the reading network. Because this was a reading task, the driving input was to the left fusiform gyrus. Syntactic complexity, however, modulated activity from the inferior frontal sulcus to the middle temporal gyrus. Findings from both DCM studies were consistent with the model proposed by Friederici (2012) and bear some similarity to the results found in the current study.

When considering the modulations induced by syntactic movement processing, the model described in Friederici (2012) explained that the backward projection from the left pars opercularis of the IFG (BA44) to the posterior superior temporal cortex is responsible for integrating semantic and syntactic information. That is, syntactic structure analysis precedes thematic role re-analysis, i.e., understanding who is doing what to whom. The left IFG, in addition, plays a role in response selection (Swick et al., 2008); in this case, comparing the semantic information expressed by the spoken sentence and the picture. It may be the case that, following integration of semantics and syntax, information is sent to the IFG in order to compare the sentential meaning to the visual information.

Within the context of the Bornkessel-Schlesewsky and Schlesewsky (2013) model, the interpretation would be that lexical-semantic and verb argument structure processing occurs in the LSTGp, followed by semantic combinatorial processes along the ventral temporofrontal pathway and syntactic combinatorial processes along the dorsal temporofrontal pathway. Pathways would converge in the LIFGop for unification of the semantic and syntactic information. However, it would then be unclear why the frontotemporal pathway is also modulated by syntactic movement.

The present results also found that connectivity between the LSFGm and LIFGop increased with both types of syntactic movement, though the role of the LSFGm is not clear. Some evidence suggests that activity in and around the LSFGm may reflect response preparation (Corbetta and Shulman, 2002; Kristensen et al., 2013) and/or cognitive control (Henry et al., 2004; Dosenbach et al., 2006). Two sentence comprehension studies previously reported activity in the LSFGm, along with the LIFG, in which the tasks involves sentence-picture matching (Kinno et al., 2008; Segaert et al., 2013). One did not provide an interpretation for LIFG activation, while the other associated it with general linguistic processing. A third sentence comprehension study found that the LSFGm was not only engaged for implausible sentences compared to plausible sentences, but also during Stroop and Flanker tasks (Ye and Zhou, 2009). Taken together, these findings suggest that the LSFGm may support domain-general cognitive processes, such as incongruence detection, as this seems to be the overarching process across the reviewed papers and present study. Within the context of the present experiment, it may be that the LSFGm is utilized for comparing the sentential meaning to the visual information, thereby allowing the participant to determine a match or mismatch response.

Finally, it should be noted that this analysis was hypothesis-driven and only included model configurations that were compatible with accounts of sentence processing supported either by Friederici (2012) or Bornkessel-Schlesewsky and Schlesewsky (2013). In other words, this study was not designed to exhaustively test all possible model configurations.

In conclusion, activation and connectivity patterns from this study were consistent with previous research supporting the model of auditory sentence comprehension posed by Friederici (2012). This model claims that processing complex sentences involves assigning grammatical relations, which is linked to the opercular part of the left IFG, followed by thematic role re-analysis, which is associated with posterior temporal cortex. Our results, however, should be taken with caution as peak fMRI activation was variable in location and strength across participants, though these factors were most consistent in the nodes of the effective connectivity models. Also, the DCM analysis did not exhaust all possible model configurations, though the model space was limited to include the most plausible model configurations according to neurolinguistics theories and to increase efficiency by optimizing computational processing time. Given these limitations, the findings from the present study suggest some greater complexity in the grammatical relationships and thematic role assignments when processing non-canonical sentences with wh-movement compared to those with NP-movement.

## Author Contributions

EE and CT worked together on the project from conceptualization to publication of the results. CT served as the primary scientific mentor to EE, and contributed significantly to the experimental design and methods, including development of behavioral and neuroimaging tasks. EE acquired and analyzed the MRI data and conducted the effective connectivity analyses under the direction of CT and with additional guidance from SK and DG. All authors contributed to the interpretation of results. EE took the lead in writing the manuscript and created the figures and tables. CT, DG, and SK provided critical feedback on all drafts of the manuscript.

### Conflict of Interest Statement

SK serves as a guest associate editor for Frontiers. The remaining authors declare that the research was conducted in the absence of any commercial or financial relationships that could be construed as a potential conflict of interest.

## Footnotes

^1^Audacity® software is copyright © 1999–2014 Audacity Team. The name Audacity® is a registered trademark of Dominic Mazzoni.
