# Supplementary material for: Neural Connectivity in Syntactic Movement Processing
Source: Front Hum Neurosci. 2019 Feb 13;13:27. doi: 10.3389/fnhum.2019.00027 (PMC6381040; doi:10.3389/fnhum.2019.00027)
Supplement: Supplementary file 1 [file Data_Sheet_1.docx]

**Appendix: Experimental Sentences**

**Neural Connectivity in Syntactic Movement Processing**

Eduardo Europa, Darren R. Gitelman, Swathi Kiran, & Cynthia K. Thompson

Frontiers in Human Neuroscience (2019), 13:27, doi: 10.3389/fnhum.2019.00027

| ACTIVE | PASSIVE |
| --- | --- |
| 1. The woman was burying the boy. | 1. The woman was buried by the boy. |
| 1. The boy was burying the woman. | 1. The boy was buried by the woman |
| 1. The man was calling the woman. | 1. The man was called by the woman. |
| 1. The woman was calling the man. | 1. The woman was called by the man. |
| 1. The girl was capturing the boy. | 1. The boy was captured by the girl. |
| 1. The boy was capturing the girl. | 1. The girl was captured by the boy. |
| 1. The man was carrying the woman | 1. The woman was carried by the man. |
| 1. The woman was carrying the man. | 1. The man was carried by the woman. |
| 1. The woman was cleaning the girl. | 1. The woman was cleaned by the girl. |
| 1. The girl was cleaning the woman. | 1. The girl was cleaned by the woman. |
| 1. The man was combing the woman. | 1. The man was combed by the woman. |
| 1. The woman was combing the man. | 1. The woman was combed by the man. |
| 1. The man was covering the woman. | 1. The man was covered by the woman. |
| 1. The woman was covering the man. | 1. The woman was covered by the man. |
| 1. The girl was examining the boy. | 1. The boy was examined by the girl. |
| 1. The boy was examining the girl. | 1. The girl was examined by the boy. |
| 1. The dog was following the cat. | 1. The dog was followed by the cat. |
| 1. The cat was following the dog. | 1. The cat was followed by the dog. |
| 1. The man was greeting the girl. | 1. The man was greeted by the girl. |
| 1. The girl was greeting the man. | 1. The girl was greeted by the man. |
| 1. The girl was hugging the man. | 1. The man was hugged by the girl. |
| 1. The man was hugging the girl. | 1. The girl was hugged by the man. |
| 1. The girl was kicking the boy. | 1. The girl was kicked by the boy. |
| 1. The boy was kicking the girl. | 1. The boy was kicked by the girl. |
| 1. The cat was licking the dog. | 1. The dog was licked by the cat. |
| 1. The dog was licking the cat. | 1. The cat was licked by the dog. |
| 1. The man was lifting the woman. | 1. The woman was lifted by the man. |
| 1. The woman was lifting the man. | 1. The man was lifted by the woman. |
| 1. The woman was observing the man. | 1. The woman was observed by the man. |
| 1. The man was observing the woman. | 1. The man was observed by the woman. |
| 1. The woman was photographing the man. | 1. The man was photographed by the woman. |
| 1. The man was photographing the woman. | 1. The woman was photographed by the man. |
| 1. The woman was pinching the man. | 1. The man was pinched by the woman. |
| 1. The man was pinching the woman. | 1. The woman was pinched by the man. |
| 1. The boy was poking the girl. | 1. The boy was poked by the girl. |
| 1. The girl was poking the boy. | 1. The girl was poked by the boy. |
| 1. The boy was pushing the girl. | 1. The boy was pushed by the girl. |
| 1. The girl was pushing the boy. | 1. The girl was pushed by the boy. |
| 1. The dog was scratching the cat. | 1. The cat was scratched by the dog. |
| 1. The cat was scratching the dog. | 1. The dog was scratched by the cat. |
| 1. The boy was shaving the man. | 1. The boy was shaved by the man. |
| 1. The man was shaving the boy. | 1. The man was shaved by the boy. |
| 1. The boy was tackling the girl. | 1. The girl was tackled by the boy. |
| 1. The girl was tackling the boy. | 1. The boy was tackled by the girl. |
| 1. The boy was tickling the girl. | 1. The boy was tickled by the girl. |
| 1. The girl was tickling the boy. | 1. The girl was tickled by the boy. |
| 1. The man was touching the woman. | 1. The woman was touched by the man. |
| 1. The woman was touching the man. | 1. The man was touched by the woman. |

| SUBJECT-CLEFT | OBJECT-CLEFT |
| --- | --- |
| 1. It was the woman who buried the boy. | 1. It was the woman who the boy buried. |
| 1. It was the boy who buried the woman. | 1. It was the boy who the woman buried. |
| 1. It was the woman who called the man. | 1. It was the man who the woman called. |
| 1. It was the man who called the woman. | 1. It was the woman who the man called. |
| 1. It was the boy who captured the girl. | 1. It was the boy who the girl captured. |
| 1. It was the girl who captured the boy. | 1. It was the girl who the boy captured. |
| 1. It was the man who carried the woman. | 1. It was the woman who the man carried. |
| 1. It was the woman who carried the man. | 1. It was the man who the woman carried. |
| 1. It was the girl who cleaned the woman. | 1. It was the girl who the woman cleaned. |
| 1. It was the woman who cleaned the girl. | 1. It was the woman who the girl cleaned. |
| 1. It was the man who combed the woman. | 1. It was the woman who the man combed. |
| 1. It was the woman who combed the man. | 1. It was the man who the woman combed. |
| 1. It was the man who covered the woman. | 1. It was the man who the woman covered. |
| 1. It was the woman who covered the man. | 1. It was the woman who the man covered. |
| 1. It was the boy who examined the girl. | 1. It was the boy who the girl examined. |
| 1. It was the girl who examined the boy. | 1. It was the girl who the boy examined. |
| 1. It was the cat who followed the dog. | 1. It was the dog who the cat followed. |
| 1. It was the dog who followed the cat. | 1. It was the cat who the dog followed. |
| 1. It was the man who greeted the girl. | 1. It was the man who the girl greeted. |
| 1. It was the girl who greeted the man. | 1. It was the girl who the man greeted. |
| 1. It was the girl who hugged the man. | 1. It was the girl who the man hugged. |
| 1. It was the man who hugged the girl. | 1. It was the man who the girl hugged. |
| 1. It was the girl who kicked the boy. | 1. It was the girl who the boy kicked. |
| 1. It was the boy who kicked the girl. | 1. It was the boy who the girl kicked. |
| 1. It was the dog who licked the cat. | 1. It was the dog who the cat licked. |
| 1. It was the cat who licked the dog. | 1. It was the cat who the dog licked. |
| 1. It was the woman who lifted the man. | 1. It was the woman who the man lifted. |
| 1. It was the man who lifted the woman. | 1. It was the man who the woman lifted. |
| 1. It was the woman who observed the man. | 1. It was the woman who the man observed. |
| 1. It was the man who observed the woman. | 1. It was the man who the woman observed. |
| 1. It was the man who photographed the woman. | 1. It was the man who the woman photographed. |
| 1. It was the woman who photographed the man. | 1. It was the woman who the man photographed. |
| 1. It was the man who pinched the woman. | 1. It was the woman who the man pinched. |
| 1. It was the woman who pinched the man. | 1. It was the man who the woman pinched. |
| 1. It was the girl who poked the boy. | 1. It was the boy who the girl poked. |
| 1. It was the boy who poked the girl. | 1. It was the girl who the boy poked. |
| 1. It was the boy who pushed the girl. | 1. It was the girl who the boy pushed. |
| 1. It was the girl who pushed the boy. | 1. It was the boy who the girl pushed. |
| 1. It was the cat who scratched the dog. | 1. It was the cat who the dog scratched. |
| 1. It was the dog who scratched the cat. | 1. It was the dog who the cat scratched. |
| 1. It was the man who shaved the boy. | 1. It was the man who the boy shaved. |
| 1. It was the boy who shaved the man. | 1. It was the boy who the man shaved. |
| 1. It was the girl who tackled the boy. | 1. It was the boy who the girl tackled. |
| 1. It was the boy who tackled the girl. | 1. It was the girl who the boy tackled. |
| 1. It was the girl who tickled the boy. | 1. It was the girl who the boy tickled. |
| 1. It was the boy who tickled the girl. | 1. It was the boy who the girl tickled. |
| 1. It was the woman who touched the man. | 1. It was the woman who the man touched. |
| 1. It was the man who touched the woman. | 1. It was the man who the woman touched. |
